# A Snu114–GTP–Prp8 module forms a relay station for efficient splicing in yeast

**DOI:** 10.1093/nar/gkaa182

**Published:** 2020-03-20

**Authors:** Junqiao Jia, Oleg M Ganichkin, Marco Preußner, Eva Absmeier, Claudia Alings, Bernhard Loll, Florian Heyd, Markus C Wahl

**Affiliations:** 1 Freie Universität Berlin, Laboratory of Structural Biochemistry, Takustraβe 6, D-14195 Berlin, Germany; 2 Freie Universität Berlin, Laboratory of RNA Biochemistry, Takustraβe 6, D-14195 Berlin, Germany; 3 Helmholtz-Zentrum Berlin für Materialien und Energie, Macromolecular Crystallography, Albert-Einstein-Straße 15, D-12489 Berlin, Germany

## Abstract

The single G protein of the spliceosome, Snu114, has been proposed to facilitate splicing as a molecular motor or as a regulatory G protein. However, available structures of spliceosomal complexes show Snu114 in the same GTP-bound state, and presently no Snu114 GTPase-regulatory protein is known. We determined a crystal structure of Snu114 with a Snu114-binding region of the Prp8 protein, in which Snu114 again adopts the same GTP-bound conformation seen in spliceosomes. Snu114 and the Snu114–Prp8 complex co-purified with endogenous GTP. Snu114 exhibited weak, intrinsic GTPase activity that was abolished by the Prp8 Snu114-binding region. Exchange of GTP-contacting residues in Snu114, or of Prp8 residues lining the Snu114 GTP-binding pocket, led to temperature-sensitive yeast growth and affected the same set of splicing events *in vivo*. Consistent with dynamic Snu114-mediated protein interactions during splicing, our results suggest that the Snu114–GTP–Prp8 module serves as a relay station during spliceosome activation and disassembly, but that GTPase activity may be dispensable for splicing.

## INTRODUCTION

Precursor messenger RNA (pre-mRNA) splicing entails the removal of non-coding introns and the ligation of neighboring coding exons and represents a key co-/post-transcriptional gene expression and gene regulatory process in eukaryotes. Splicing is mediated by the spliceosome, an elaborate RNA-protein (RNP) molecular machine that encompasses five small nuclear (sn) RNPs (U1, U2, U4, U5 and U6 in the case of the major spliceosome) and many non-snRNP factors (1,2). Each U snRNP contains a unique snRNA, a set of seven common Sm or, in the case of U6, Sm-like (LSm) proteins and a varying number of particle-specific proteins (3). For every splicing event, a spliceosome is assembled *de novo* from component subunits, catalytically activated and disassembled after the splicing reaction (1,2). Almost all of the comparatively small number of intron-containing genes in yeast harbor a single intron, and the resulting pre-mRNAs are spliced constitutively (1). In contrast, most genes in higher eukaryotes contain more than one intron and their pre-mRNAs can be spliced in a flexible manner, giving rise to different mature mRNAs that contain different combinations of exons (alternative splicing) (4).

Transitions between functional stages of a splicing cycle are accompanied by massive compositional and conformational remodeling of the underlying spliceosomal RNP interaction networks (1–2,5–6). Constitutive splicing events in yeast follow a canonical cross-intron spliceosome assembly pathway that is initiated by U1 snRNP recognizing the 5′-splice site (SS), splicing factor 1 (SF1) binding a conserved branch point sequence in the intron and the U2 auxiliary factors (U2AF) 1/2 recognizing a poly-pyrimidine tract and the 3′SS, respectively, forming the E-complex. Subsequently, U2 snRNP replaces SF1 at the branch point sequence, giving rise to complex A. The remaining three snRNPs then join as a pre-formed U4/U6•U5 tri-snRNP to yield the pre-B and, after release of U1 snRNP, the B complex. After disruption of the initially base-paired U4/U6 di-snRNAs, displacement of U4 and U4/U6-associated proteins and concomitant recruitment of the non-snRNP NineTeen complex (NTC), the ensuing activated spliceosome (B^act^ complex) is further rearranged to form the catalytically activated spliceosome (catalytic pre-branching B* complex), which carries out the first step of splicing. Remodeling of the resulting catalytic post-branching complex C yields the catalytic pre-exon ligation complex C*, which mediates the second transesterification step. The ensuing post-splicing P complex releases the mRNA product as an mRNP, giving rise to the intron-lariat spliceosome (ILS), from which the remaining subunits are recycled.

The spliceosomal assembly, activation, catalysis and disassembly cycle is driven and controlled by eight highly conserved superfamily 2 RNA-dependent NTPases/RNA helicases and a single G protein, Snu114 (7,8). While specific functions have by now been attributed to the NTPases, the role of the Snu114 GTPase remains enigmatic. Snu114 bears striking resemblance to the prokaryotic/eukaryotic ribosomal translocases EF-G/eEF2, exhibiting the same five-domain arrangement preceded by a Snu114-specific, ca. 125 residue, acidic N-terminal region (9). Removal of the N-terminal region or mutations in other regions of Snu114 in yeast led to a block in splicing before the first catalytic step (10,11), implicating the protein in spliceosome activation. Consistent with this notion and with GTP hydrolysis by Snu114 being important for this process, a D271N mutation in the G domain of Snu114, which renders the protein XTP-specific, also led to a block of spliceosome activation, which was partially overcome by addition of XTP and ATP (12). Furthermore, mutations in all EF-G/eEF2-like domains have been identified that exhibit growth defects, led to accumulation of pre-catalytic spliceosomes and/or showed genetic interactions with factors involved in snRNP biogenesis, snRNP stability, B complex formation or spliceosome activation (11–13). Moreover, mutations in the G domain of Snu114 led to U5 snRNP and U4/U6•U5 tri-snRNP assembly defects (12,13). Based on these studies and the similarities to EF-G/eEF2, Snu114 has been proposed to act as a mechano-chemical motor that drives RNA–RNA or RNA-protein rearrangements in the spliceosome (7,11–12). Snu114 has also been implicated in spliceosome disassembly (14). However, while spliceosome activation and disassembly seem to require GTP-bound Snu114, they did not depend on GTP hydrolysis, suggesting that Snu114 may rather act like a classic regulatory G protein that controls the activity of the spliceosomal helicase Brr2 depending on its nucleotide-bound state (14).

Based on the latter findings, spliceosomal Snu114 regulatory factors, such as a GTPase activating protein (GAP), a guanine nucleotide exchange factor (GEF) and/or a guanine nucleotide dissociation inhibitor (GDI), have been postulated (14), but presently the identity of such putative regulators is unclear. A prime candidate for such functions is the Prp8 protein, which forms a salt-stable complex with Snu114 (15), extensively interacts with Snu114 G and G’ domains in structures of spliceosomal complexes (16,17) and is generally considered a master regulator of the spliceosome (18). Here, we have determined the crystal structure of yeast Snu114 in complex with an N-terminal fragment of Prp8 (Prp8 Snu114-binding region, Prp8^SBR^) and GTP. Biochemical analyses showed that Prp8^SBR^ completely abrogated the very low intrinsic GTPase activity of Snu114. Based on the structure, we identified Snu114 and Prp8 mutations that led to yeast growth defects and affect splicing of the same sub-set of genes in yeast. Our results suggest that stable Snu114 GTP binding supported by Prp8^SBR^ is essential for splicing of at least a subset of pre-mRNAs, but that Snu114-mediated GTP hydrolysis may not be required for splicing.

## MATERIALS AND METHODS

### Cloning, expression and protein purification

The MultiBac system (19) was used to express proteins of interest in insect cells. Synthetic genes encoding yeast Snu114^72-1008^, Prp8^132-2413^ and Aar2 were cloned into modified pIDS-C-Strep, pFL-C-Strep and pIDK-C-Strep vectors, respectively. After Cre-mediated recombination of the three plasmids, virus and the Snu114^72-1008^–Prp8^132-2413^–Aar2 complex were produced in insect cells as described previously (20). A DNA fragment encoding yeast Prp8^SBR^ was sub-cloned to pFL-N-Strep vector. pIDS-C-Strep-*SNU114*^72-1008^ and pFL-N-Strep-*PRP8*^SBR^ were Cre-recombined and used to produce virus and the Snu114^72-1008^–Prp8^SBR^ complex in insect cells as described before (20). Site-directed mutagenesis was performed using the QuikChange II XL Site-Directed Mutagenesis Kit (Agilent). All constructs were confirmed by Sanger sequencing.

For purification of yeast Snu114^72-1008^–Prp8^132-2413^–Aar2 complex, the cell pellet was re-suspended in lysis buffer I (20 mM HEPES-NaOH, pH 8.0, 400 mM NaCl, 2 mM DTT, 5% (v/v) glycerol, 0.03% (v/v) Triton X-100) supplemented with DNase I, RNase A and avidin (Thermo Fisher Scientific) to final concentrations of 0.01 mg/ml, 0.1 mg/ml and 0.01 mg/ml, respectively. Cells were lysed by sonication using a Sonopuls Ultrasonic Homogenizer (Bandelin), cleared by centrifugation and the supernatant was loaded on a 5 ml StrepTactin sepharose column (GE Healthcare). After washing with lysis buffer, the protein complex was eluted using lysis buffer supplemented with 2.5 mM D-desthiobiotin (Iris Biotech GmbH). The eluted protein complex was passed through 5 ml Heparin sepharose and Mono Q columns (GE Healthcare). The pooled protein complex was further purified by SEC on a Superose 6 16/300GL column (GE Healthcare) in 50 mM TRIS–HCl, pH 8.5, 300 mM NaCl, 2 mM DTT.

For purification of yeast Snu114^72-1008^–Prp8^SBR^ complex, the cell pellet was re-suspended in lysis buffer II (50 mM TRIS–HCl, pH 8.0, 300 mM NaCl, 2 mM DTT, 0.05% (v/v) Triton X-100) supplemented with protease inhibitors (Roche) and avidin, and lysed by sonication. After centrifugation and filtration, the extract was mixed with StrepTactin sepharose beads (Iris Biotech GmbH) in a gravity flow column and incubated for 1 hour at 4°C. After washing with lysis buffer II, the complex was eluted using lysis buffer II containing 2.5 mM D-desthiobiotin and subjected to SEC on a Superdex S200 16/600 (GE Healthcare) in 10 mM TRIS-HCl, pH 8.0, 300 mM NaCl, 2 mM DTT.

For purification of isolated yeast Snu114^72-1008^, cells were lysed in lysis buffer III (100 mM TRIS–HCl/100 mM Na-citrate, pH 6.8, 100 mM KCl, 5 mM MgCl_2_, 2 mM DTT) supplemented with protease inhibitors and avidin. The protein was captured on StrepTactin sepharose beads and eluted in 100 mM Na-citrate, pH 5.9, 100 mM KCl, 5 mM MgCl_2_, 2 mM DTT, 2.5 mM D-desthiobiotin. Pooled fractions were subjected to SEC on a Superdex S200 16/600 column in 100 mM Na-citrate, pH 5.9, 100 mM KCl, 5 mM MgCl_2_, 2 mM DTT.

### Limited proteolysis

A total of 100 μg of purified Snu114^72-1008^–Prp8^132-2413^–Aar2 complex in 40 μl buffer (50 mM TRIS–HCl, pH 8.0, 300 mM NaCl, 2 mM DTT) were incubated with 10 μl chymotrypsin at 0.05 μg/μl at room temperature for 35 min, and the reaction was stopped by adding PMSF to a final concentration of 2 mM. The sample was separated by SEC on a Superose 6 Increase 3.2/300 column (GE Healthcare) in 20 mM TRIS–HCl, pH 8.5, 500 mM NaCl, 2 mM DTT. Half of the eluted fractions were inspected by sodium dodecyl sulphate-polyacrylamide gelelectrophoresis (SDS-PAGE), and bands of interest were analyzed by tryptic mass spectrometric fingerprinting. The remainder of the fractions was separated by SDS-PAGE and blotted on a PVDF membrane, stained with Ponceau S and fragments of interest were subjected to N-terminal sequencing.

### Crystallographic analysis

For crystallization, the Snu114^72-1008^–Prp8^SBR^ complex was concentrated to 26 mg/ml. Crystallization was conducted by sitting-drop vapor diffusion in 24-well plates. The best crystals grew upon mixing 1 μl of protein solution with 1 μl of reservoir solution containing 50 mM MgSO_4_, 200 mM LiCl, 10% (w/v) PEG 8000. For cryo-protection, the crystals were transferred to reservoir solution supplemented with 20% (v/v) ethylene glycol, and flash-cooled in liquid nitrogen.

Diffraction data were collected on beamline 14.2 of the BESSY II storage ring (Berlin, Germany) at 100 K. All data were processed with XDS (21,22). The structure was solved by molecular replacement, using the coordinates of Snu114 derived from the cryo-electron microscopy (cryoEM) structure of a yeast spliceosome (PDB ID: 3JB9) (16). The structure was refined by alternating rounds of model building in Coot (23) and automated maximum-likelihood restrained refinement in PHENIX (24). Model quality was evaluated with MolProbity (25). Figures were prepared using PyMOL (26). Data collection and refinement statistics are provided in Table 1.

**Table 1.** Crystallographic data

Data collection	
Wavelength [Å]	0.91841
Space group	C2
Unit cell parameters	
a, b, c [Å]	173.1, 158.6, 110.7
β [°]	116.5
Resolution [Å]^a^	50–3.1 (3.18–3.10)
Reflections	
Total	207 277 (15 200)
Unique	48 253 (3 522)
Multiplicity	4.3 (4.3)
Completeness [%]	99.3 (99.2)
Mean I/σ(I)	12.32 (0.92)
R_merge_(I) [%]^(b)^	9.7 (151.7)
R_meas_(I) [%]^(c)^	11.0 (173.1)
CC_1/2_ [%]^(d)^	99.8 (51.6)
**Refinement**	
Resolution [Å]^a^	47.5–3.1 (3.16–3.10)
Reflections	
Unique	48 232 (4 752)
Test set [%]	5.0 (5.0)
*R*_work_ [%]^(e)^	23.0 (47.7)
*R*_free_ [%]^(f)^	27.1 (50.4)
Contents of A.U.^(g)^	
Non-H atoms	16 330
Protein residues/atoms	2 025/16 249
GTP, Mg^2+^, SO_4_^2−^ atoms	76
Water oxygens	5
Mean B factors [Å^2^]	
Wilson	87.2
Model atoms	102.3
Rmsd^(h)^ from ideal geometry	
Bond lengths [Å]	0.003
Bond angles [°]	0.74
Model quality^(i)^	
Overall score	1.98
Clash score	12.1
Ramachandran favored [%]	94.3
Ramachandran outliers [%]	0.8
PDB ID	6TEO

^a^Values in parentheses refer to the highest resolution shells.

^b^ *R*_merge_(I) = ∑_h_ ∑*_i_* │I*_i_*_h_ − <I_h_>│ / ∑_h_∑_i_ I*_i_*_h_, in which <I_h_> is the mean intensity of symmetry-equivalent reflections h and I*_i_*_h_ is the intensity of a particular observation of h (46).

^c^ *R*_meas_(I) = ∑_h_ [N/(N-1)]^1/2^ ∑_i_ │I*_i_*_h_ − <I_h_>│ / ∑_h_∑_i_ I*_i_*_h_, in which <I_h_> is the mean intensity of symmetry-equivalent reflections h, I*_i_*_h_ is the intensity of a particular observation of h and N is the number of redundant observations of reflection h (46).

^d^CC_1/2_ = (<I^2^> − <I>^2^) / (<I^2^> − <I>^2^) + σ^2^_ϵ_, in which σ^2^_ϵ_ is the mean error within a half-dataset (46).

^e^ *R*_work_ = ∑_h_ │F_o_ − F_c_│ / ∑ F_o_ (working set, no σ cut-off applied).

^f^ *R*_free_ is the same as R_work_, but calculated on the test set of reflections excluded from refinement.

^g^A.U.—asymmetric unit.

^h^Rmsd—root-mean-square deviation.

^h^Calculated with MolProbity (25).

### Analysis of Snu114-bound nucleotides

A total of 50 μl of purified Snu114^72-1008^ or -Prp8^SBR^ complex variants at 50 μM were incubated for 3 min at 95°C, centrifuged at 17 000 *g* for 5 min, and 20 μl of the supernatants were loaded on a Poroshell 120 EC-C18 RP-HPLC column (Agilent), equilibrated in 100 mM K_2_HPO_4_/KH_2_PO_4_, pH 6.5, 10 mM tetrabutylammonium bromide, 7.5% (v/v) acetonitrile. The samples were chromatographed at 1.5 ml/min. Sample buffer, GDP and GTP served as references.

### GTPase assays

To monitor steady-state GTPase, 10 μl of purified Snu114^72-1008^ or -Prp8^SBR^ complex variants at 10 μM were incubated in 25 mM TRIS–HCl pH 8.0, 300 mM NaCl, 5 mM MgCl_2_, 5 mM DTT, 12 mM [α-^32^P] GTP (6000 mCi/mmol) at 30°C for up to 60 min. Reactions were terminated at various time points by adding 10 μl of 40% formic acid, and 0.5 μl of each sample were spotted on a thin-layer chromatography (TLC) plate and dried. TLC plates were placed in a TLC chamber with 0.5 M LiCl in 80% (v/v) 1 M acetic acid/20% (v/v) ethanol. Developed TLC plates were air-dried and used to expose phosphorimager screens for 3 h. Screens were scanned on a Storm phosphorimager (GE Healthcare), and spots corresponding to GDP and GTP were quantified using ImageQuant software (GE Healthcare).

### Yeast growth assays

Yeast growth was assessed after plasmid shuffling. In the YPF8 strain (MATα, *trp1-Δ1*; *his3-Δ*; *ura3-52*; *lys2-801*; *ade2-101*; *snu114Δ::HIS3* [pRS316/*SNU114*, *ARS*, *CEN6*, *URA3*]) (9), the chromosomal *SNU114* gene is replaced by a *HIS3* marker, and the strain carries a wild-type (wt) *SNU114* gene on a counter-selectable pRS316 plasmid with a *URA3* marker. YPF8 cells were transformed with wt *SNU114* or mutant *snu114* genes on plasmid pRS314 (*TRP1*), and transformants were selected on complete minimal (CM)-agar plates lacking histidine, tryptophan and uracil. In the JDY8.06 strain (*ura3-52*; *leu2-3*,*-112*; *ade2*; *his3-A1*; *trpl-289*; *prp8::LEU2* [pY8500/*PRP8*, *ARS*, *CEN*, *URA3*]) (27), the chromosomal *PRP8* gene is replaced by a *LEU2* marker, and the strain carries a wt *PRP8* gene on a counter-selectable pY8500 plasmid with *URA3* marker. JDY8.06 cells were transformed with wt *PRP8* or mutant *prp8* genes on plasmid pJU186 (*HIS*), and transformants were selected on CM-agar plates lacking histidine, leucine and uracil. Five colonies of each were picked and streaked out on 5-fluoro-orotic acid (5-FOA; 0.1% [w/v]) CM-agar plates lacking histidine and tryptophan or histidine and leucine. Mutations in the *snu114* or *prp8* genes of the colonies growing on 5-FOA were confirmed by colony polymerase chain reaction (PCR) and sequencing. Strains that survived 5-FOA selection and carried the desired *snu114* or *prp8* mutations were grown overnight in CM medium, diluted to an OD_600_ of 1.0 and 5 μl of serial dilutions (1:1, 1:10, 1:100, 1:1000) were spotted on CM-agar plates lacking histidine and tryptophan or histidine and leucine, followed by incubation at 18°C, 30°C or 37°C for 4 days.

### *In vivo* splicing assays

Relevant yeast strains were grown in 5 ml of CM medium for 24 h, diluted to OD_600_ of 0.1 in fresh CM medium and grown at 30 or 37°C to an OD_600_ of 2.5. Cells were harvested by centrifugation (10 min, 3500 × *g*, 4°C). Cell pellets were re-suspended in denaturing solution (4 M guanidine thiocyanate, 0.5% (w/v) sarkosyl, 750 mM Na-citrate, pH 7.0, 100 mM β-mercaptoethanol). Total RNA was phenol/chloroform/isoamyl alcohol-extracted, precipitated with isopropanol and pelleted by centrifugation (1 h, 14 000 rpm, room temperature). Extraction and precipitation were repeated once and RNA was washed with 75% (v/v) ethanol. The RNA pellet was dissolved in water and treated with DNase I at 37°C for 2 h. After another round of precipitation and washing, the RNA was dissolved in water at 1 mg/ml.

Specific cDNA libraries were produced by reverse transcription with primers pairing in the 3′-exons of *TEF4*, *ERV1*, *ACT1*, *SEC17*, *NSP1*, *UBC5*, *BET1*, *HMRA*, *CIN2*, *DBP2* or*HOP2* (pre-)mRNAs (Supplementary Table S1). qRT-PCR was performed using the ABsolute QPCR SYBR Green Mix (Thermo Fisher Scientific) on a Mx3000P thermo-cycler (Stratagene) according to the manufacturer's instructions. For qRT-PCR, one or two forward primers pairing to the intron or the 5′-exon and reverse primers pairing to the intron or the 3′-exon were employed (Supplementary Table S1). All experiments were performed as biological triplicates, qRT-PCRs were performed as technical duplicates. Intron retention ratios were calculated as the amount of intron-containing transcripts versus the amount of all transcripts, normalized to the wt.

## RESULTS

### Structure of a Snu114–Prp8^SBR^ complex

**Figure 1.**
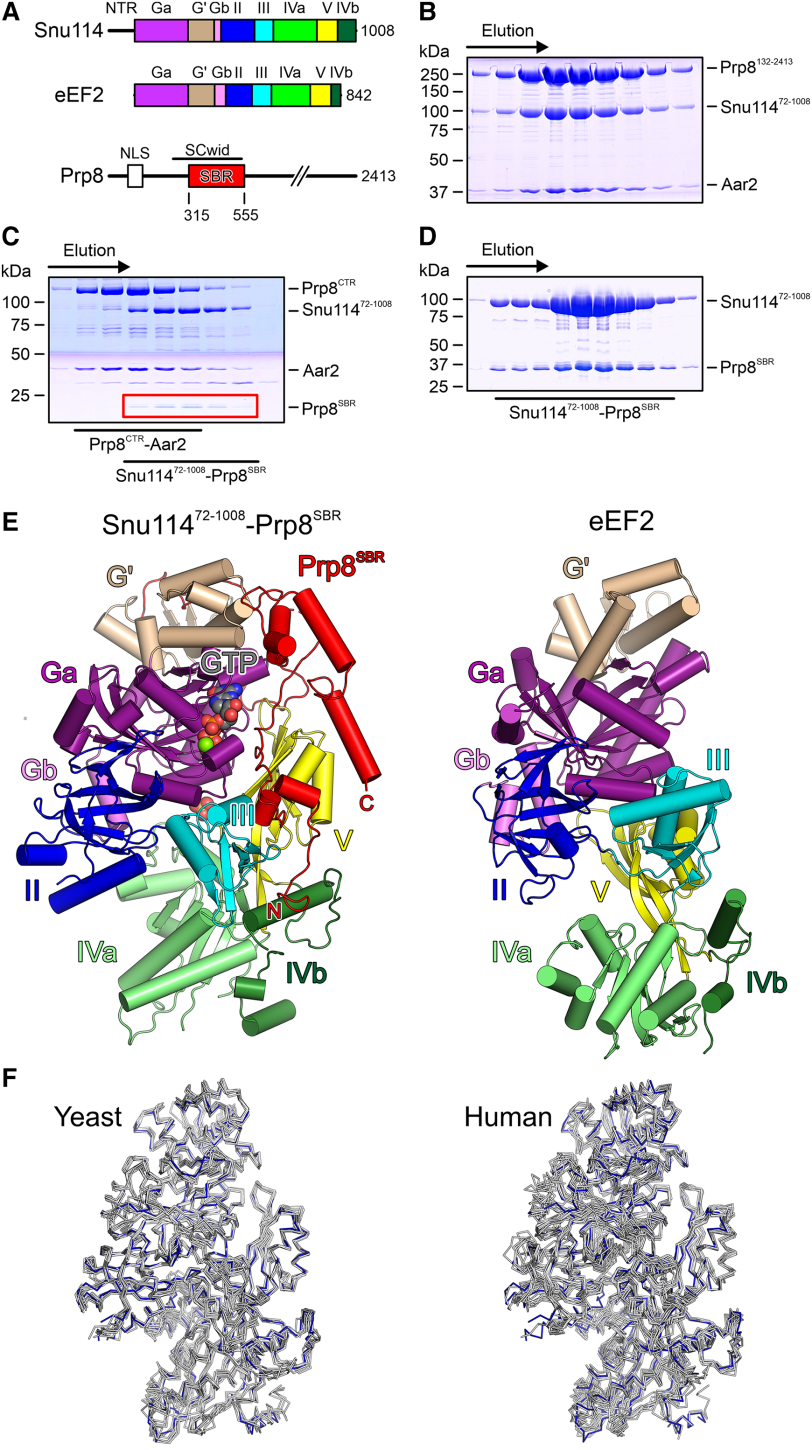
Structural overview. (**A**) Domain organization of yeast Snu114, eEF2 and the N-terminal portion of Prp8 (drawn to scale). NTR, N-terminal region; Ga, first part of G domain, G’, G’ domain; Gb, second part of G domain; II/III/V, domains II/III/V; IVa, first part of domain IV; IVb, second part of domain IV; NLS, nuclear localization signal; SCwid, Snu114/Cwc21-interaction domain; SBR, Snu114-binding region. Domain coloring is maintained in the following figures. (**B**) SDS-PAGE analysis of an analytical gel filtration run of a recombinant Prp8^132-2013^–Snu114^72-1008^–Aar2 complex. (**C**) SDS-PAGE analysis of an analytical gel filtration run of chymotrypsin-treated, recombinant Prp8^132-2013^–Snu114^72-1008^–Aar2 complex. Elution positions of two sub-complexes (Prp8^CTR^–Aar2 and Snu114^72-1008^–Prp8^SBR^) are indicated below the gel. CTR, C-terminal region. (**D**) SDS-PAGE analysis of an analytical gel filtration run of a recombinant Snu114^72-1008^–Prp8^SBR^ complex. (**E**) Comparison of the overall structures of a Snu114^72-1008^–GTP–Prp8^SBR^ complex (left) and of yeast eEF2 (right; PDB ID: 1N0V; (47)) after superposition of the G domains. Proteins are shown as cartoons with helices as cylinders and sheets as arrows. Mg^2+^-GTP in the Snu114^72-1008^–GTP–Prp8^SBR^ complex is shown as spheres colored by atom type. Carbon, gray; nitrogen, blue; oxygen, red; phosphorus, orange; magnesium, green. N/C, N-/C-termini of Prp8^SBR^. (**F**) Ribbon plots comparing the conformation of Snu114^72-1008^ in the isolated Snu114^72-1008^–GTP–Prp8^SBR^ complex (blue) with corresponding Snu114 regions in structures of yeast (left) or human (right) spliceosomal complexes. Yeast complexes/PDB IDs/references: U4/U6•U5 tri-snRNP/5GAN/(17); pre-B complex/5ZWM/(48); B complex/5ZWO/(48); B^act^ complex/5GM6/(49); B* complex/6J6G/(50); C complex/5GMK/(51); C* complex/5WSG/(52); P complex/6BK8/(53); ILS/5Y88/(54). Human complexes/PDB IDs/references: U4/U6•U5 tri-snRNP/6Q6W/(55); pre-B complex/6QX9/(55); B complex/6AHD/(56); B^act^ complex/6FF4/(57); C complex/5YZG/(58); C* complex/5MQF/(59); P complex/6ICZ/(60); ILS/6ID1/(60).

Snu114 adopts virtually identical, GTP-bound conformations in all cryoEM structures of spliceosomes and of U4/U6•U5 tri-snRNPs available to date. As Snu114 bears close resemblance to the ribosomal translocases, EF-G/eEF2 (Figure 1A), which undergo large-scale conformational changes upon binding to ribosomes, and as the Snu114 GTPase has been implicated in U5 snRNP or U4/U6•U5 tri-snRNP assembly (12,13), we wondered whether a wider conformational spectrum may be accessible to the protein outside its spliceosomal environments. However, we were unable to crystallize isolated, full-length, recombinant yeast Snu114 or a deletion variant lacking the intrinsically unstructured, acidic, N-terminal region (Snu114^72-1008^). We thus turned to a Snu114–Prp8–Aar2 complex, comprised of Snu114^72-1008^, a large fragment of the Prp8 protein lacking the first 131 residues (Prp8^132-2413^) and Aar2. Aar2 is a U5 snRNP assembly factor in yeast, which associates with pre-U5 snRNP particles during U5 snRNP biogenesis in the cytoplasm and is replaced by the Brr2 RNA helicase in mature U5 in the nucleus (28–32). To experimentally delineate interacting regions from binding partners that may aid in crystallization, we subjected this trimeric complex, which we obtained by co-production of the proteins *via* a recombinant baculovirus in insect cells, to limited proteolysis (Figure 1B and C). Chymotrypsin treatment gave rise to two sub-complexes, a large C-terminal region of Prp8 (Prp8^CTR^) that remained stably bound to Aar2 and a region encompassing Prp8 residues 315–555 (Prp8 Snu114-binding region, Prp8^SBR^) that formed a complex with Snu114^72-1008^, as revealed by mass spectrometric fingerprinting and N-terminal sequencing (Figure 1C). Prp8^CTR^-Aar2 most likely corresponds to a complex of the two proteins whose structure has previously been determined (32). Prp8^SBR^ is largely congruent with the N-terminal three quarters of a Prp8 element previously characterized as a Snu114/Cwc21-interacting domain (SCwid; Prp8 residues 253–543) (33).

Based on these observations, we prepared a recombinant Snu114^72-1008^–Prp8^SBR^ complex by co-production of Snu114^72-1008^ and Prp8^SBR^ in insect cells (Figure 1D), and determined its crystal structure at 3.1 Å resolution. Crystals contained two independent copies of the Snu114^72-1008^–Prp8^SBR^ complex, which were virtually identical (root-mean-square deviation [rmsd] of 0.66 Å for 997 pairs of Cα atoms). The following discussion refers to both complexes in a crystallographic asymmetric unit. For Snu114^72-1008^, we could trace residues 103–1008, with regions 72–102, 520–531, 691–704 and 977–984 lacking interpretable electron density. The modeled portion of Prp8^SBR^ encompasses residues 367–533, with 52 N-terminal, 26 C-terminal and internal residues 416–419 and 435–449 lacking clear electron density.

**Figure 2.**
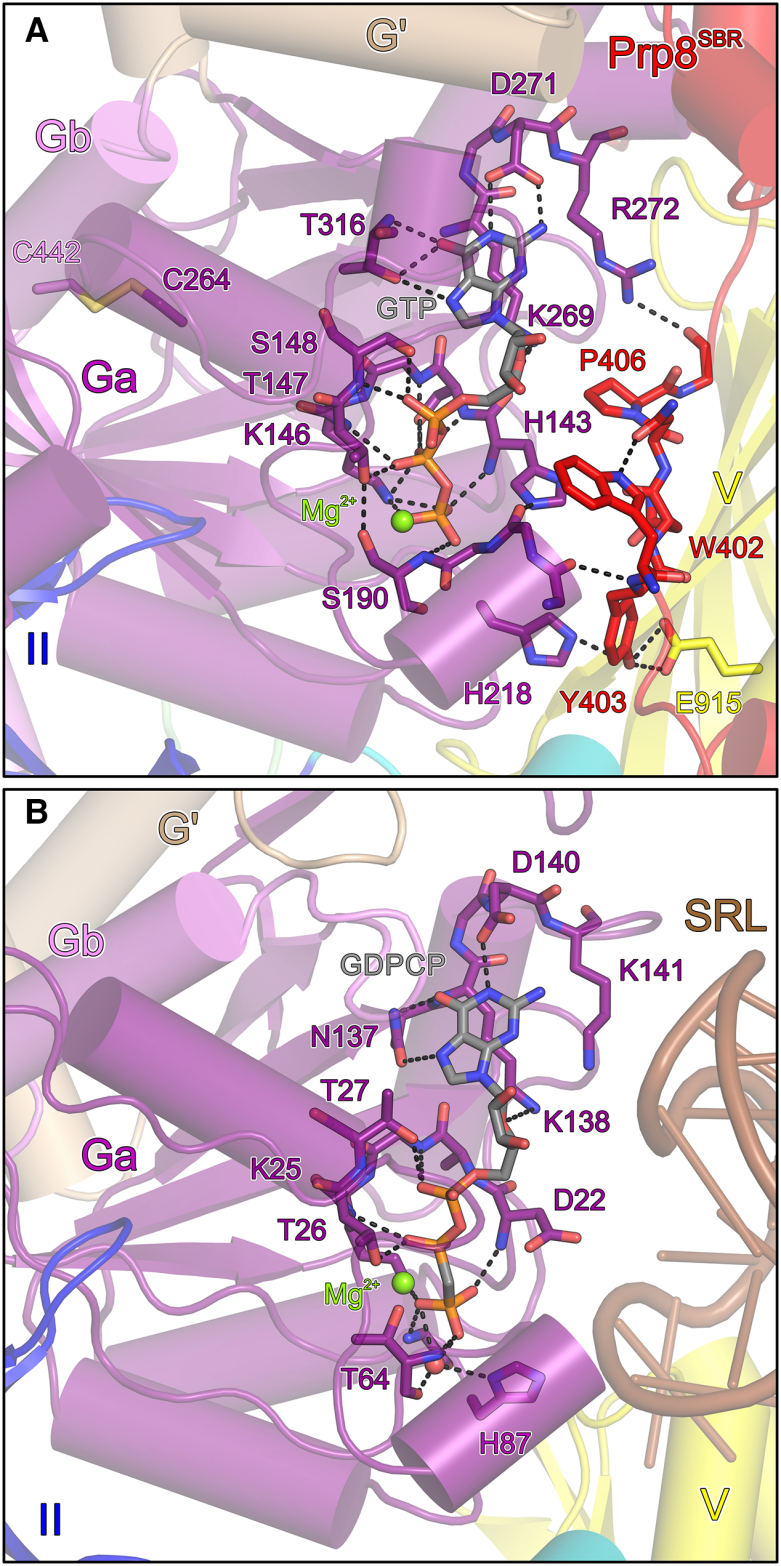
GTP binding pocket. (**A**) Mg^2+^-GTP bound at Snu114^72-1008^ in complex with Prp8^SBR^. (**B**) Mg^2+^-GDPCP bound at EF-G on the ribosome (PDB ID: 4WPO; (34)). Domains are labeled as in Figure 1A. GTP and relevant protein residues are shown as sticks colored by atom type (as in Figure 1E; except carbon, as the respective protein/domain). Green spheres, Mg^2+^ ions. Dashed lines, hydrogen bonds or salt bridges. SRL, sarcin-ricin loop.

Snu114^72-1008^ contains five EF-G/eEF2-like domains, i.e. G, G’, II, III, IV and V, with the G’ domain inserted into the G domain, and domain V intervening between the IVa and IVb sub-domains (Figures 1A and E). It adopts a compact conformation, in which the N-terminal G and G’ domains form a globular head that is cradled in an array of domains II, III and V, and in which the split domain IV forms a pedestal at the bottom (Figure 1E). Cys264 and Cys442 in the G domain of one Snu114^72-1008^ molecule in an asymmetric unit are partially engaged in a disulfide bridge that links the N- and C-terminal parts of the G domain (Figure 2A); the corresponding disulfide bridge is broken in the other Snu114^72-1008^ molecule, most likely due to radiation damage. Prp8^SBR^ exhibits an extended, loosely twisted conformation that lacks a globular fold, suggesting that the fragment would be intrinsically disordered in isolation (Figure 1E). The N-terminus of Prp8^SBR^ resides on one side of the IVb sub-domain of Snu114. The protein then meanders along the bottom part of domain V and along domain III toward the G nucleotide-binding pocket of the Snu114 G domain. Residues 402–410 stretches along the open side of the nucleotide-binding pocket (Figure 2A) and the following residues 411–471 form a long loop, previously referred to as the Prp8 lasso (16), which encircles a protruding region of the G’ domain (Figure 1E). Prp8^SBR^ then returns toward domain V of Snu114, forming a small helical bundle at one tip of the G’ domain and ends in an α helix that runs along the top of domain V (Figure 1E). The C-terminal 43 residues of Prp8^SBR^ adopt a different conformation in the U4/U6•U5 tri-snRNP and in spliceosomes due to alternative interactions with other parts of Prp8 (Supplementary Figure S1). Whether the conformation seen in the present crystal structure is an artifact due to the crystallization of a proteolyzed fragment or whether it may exist in another context, such as during U5 snRNP assembly, remains to be seen.

Contrary to our initial expectation, Snu114^72-1008^ in complex with Prp8^SBR^ closely resembles the structures of the corresponding Snu114 regions in available cryoEM structures of yeast or human spliceosomal complexes, (rmsd values of about 1 Å for about 900 pairs of Cα atoms; Figure 1F). Thus, Snu114^72-1008^ in complex with Prp8^SBR^ seems to represent a rigid structural building block of the spliceosome.

### Prp8^SBR^ stabilizes Snu114^72-1008^ in a non-hydrolytic conformation

In the crystal structure of the isolated Snu114^72-1008^–Prp8^SBR^ complex, the electron density clearly indicated that both Snu114 molecules in an asymmetric unit were bound to GTP and a metal ion, most likely Mg^2+^, although no nucleotide had been added during purification or crystallization. Mg^2+^-GTP is bound in a similar manner as seen for Mg^2+^-GDPCP bound to the bacterial ribosomal translocase EF-G on the 70S ribosome in the pre-translocated state (34) (Figure 2A and B). G proteins employ up to five conserved sequence motifs (G1–G4) for G nucleotide binding (35). In Snu114, the Watson–Crick flank of the nucleobase is recognized by the side chains of D271 (G4 motif) and T316 (G5 motif), and T316 also forms a hydrogen bond with the N7 position of GTP. The ribose O4 is bound by K269 (G4 motif). The phosphate groups are predominantly recognized by residues from the G1 motif/P-loop, with the backbone NH groups of H143, S144, G145, T147 and S148 forming hydrogen bonds to the α, β and γ-phosphates. Additionally, the S144 side chain hydrogen bonds with the β-phosphate, the side chain of K146 engages in ionic interactions with the β and γ-phosphate groups, the side chain hydroxyl of T147 hydrogen bonds with the β-phosphate and the side chain of S148 forms a hydrogen bond with the α-phosphate. The γ-phosphate is additionally hydrogen-bonded to the backbone NH group G217 (G3 motif/switch 2) as well as the backbone NH of S190 (G2/switch 1). The Mg^2+^ ion is coordinated by the β and γ-phosphates, as well as by the side chains of T147 (G1 motif/P-loop) and S190 (G2 motif/switch 1).

Previous studies had established that a conserved histidine in translation factor GTPases (H87 in EF-G) serves to position and polarize a catalytic water molecule for GTP hydrolysis (36). On the ribosome, EF-G H87 is brought into its hydrolysis-supporting conformation by the sarcin/ricin loop, a conserved element of 23S ribosomal RNA that forms part of the ribosome's GTPase-activating center (Figure 2B). H218 is the equivalent residue in Snu114. Similar to the situation in structures of spliceosomal complexes (17), H218 in the isolated Snu114^72-1008^–Prp8^SBR^ complex is rotated away from the GTP γ-phosphate, hydrogen bonding with the hydroxyl group of Prp8^SBR^ Y403 (Figure 2A). W402 of Prp8^SBR^ lies on top of the Snu114^72-1008^ switch I region, helping to anchor the neighboring Prp8^SBR^ Y403 in front of the nucleotide-binding pocket. The conformation of Y403 is additionally stabilized by E915 from domain V of Snu114^72-1008^ (Figure 2A) Thus, Prp8^SBR^ may stabilize a non-hydrolytic conformation in Snu114^72-1008^.

### Prp8^SBR^ stabilizes GTP-bound Snu114^72-1008^ and inhibits its low, intrinsic GTPase activity

To investigate the importance of Snu114^72-1008^ and Prp8^SBR^ residues in stable GTP anchoring, we used structure-guided, site-directed mutagenesis to alter residues in Snu114^72-1008^ or the Snu114^72-1008^–Prp8^SBR^ complex that potentially affect Snu114^72-1008^–GTP or Snu114^72-1008^–Prp8^SBR^ interactions. While isolated wt Snu114^72-1008^ could be produced soluble in insect cells and purified, all tested Snu114^72-1008^ variants (H218A, S190A, K146A, T147V, E915Q/A/D) formed insoluble aggregates when produced alone in insect cells. This observation is consistent with the idea that the affected residues are required for stable GTP binding to Snu114^72-1008^ and that bound GTP is required to maintain a stable fold in Snu114^72-1008^. Interestingly, several of the tested Snu114^72-1008^ variants (K146A, S190A) could be produced in soluble form together with Prp8^SBR^, indicating that Prp8^SBR^ stabilizes the fold of Snu114^72-1008^. Similarly, Prp8^SBR^ variants (wt, Y403A, Y403F, WY402-403AA, Δ402–406, 402–406_5S and Δ421–468) could not be produced alone, but could be made in complex with Snu114. Thus, Snu114 presumably protects Prp8^SBR^ and variants from degradation.

**Figure 3.**
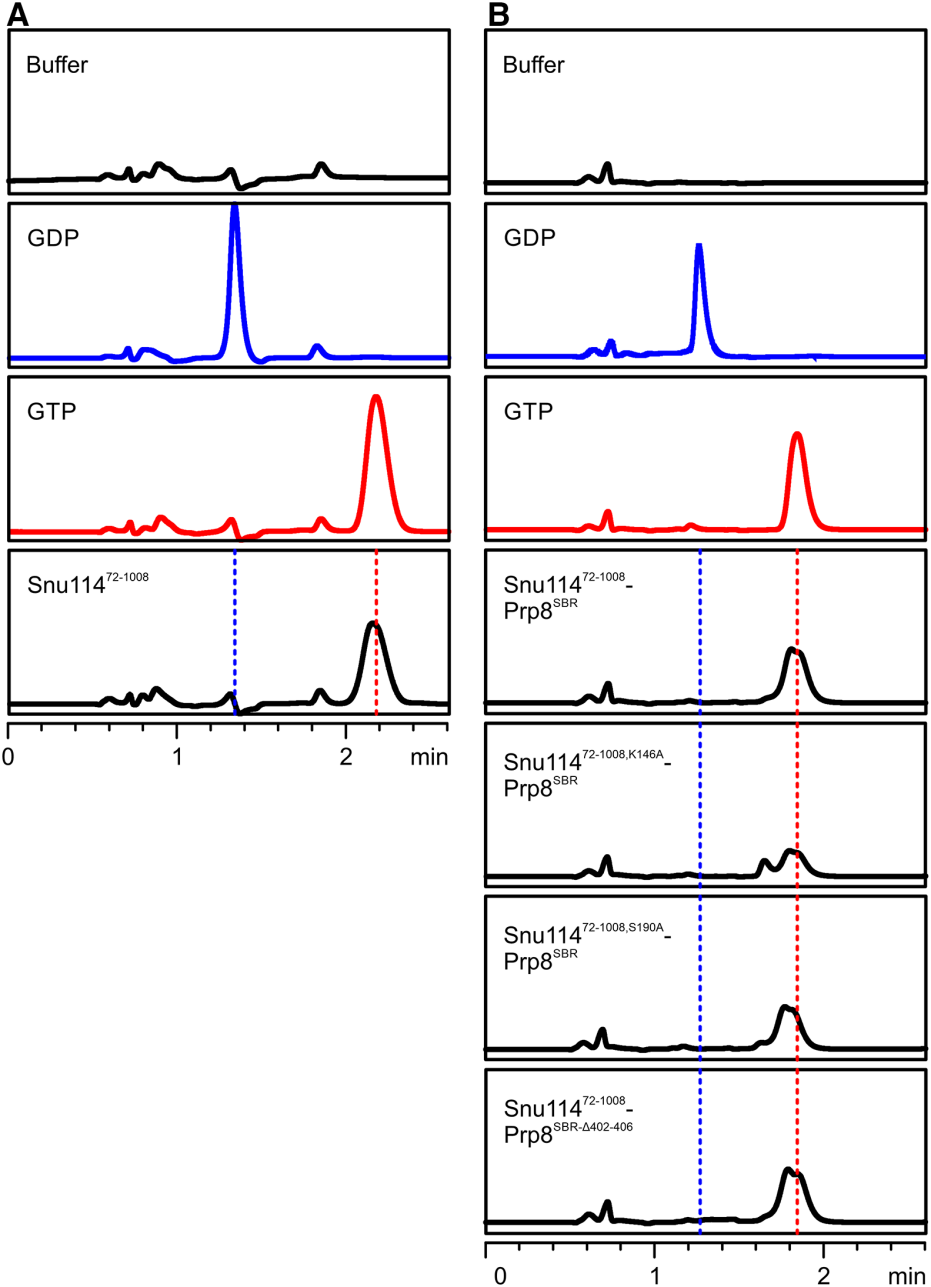
Snu114-bound G nucleotides. (**A** and**B**) RP-HPLC analysis of nucleotides bound to Snu114^72-1008^ (A) and to the indicated Snu114^72-1008^–Prp8^SBR^ complexes (B). Snu114^72-1008^ and all complexes co-purify with GTP. Buffer, GDP (blue) and GTP (red) control runs are shown on the top. Different retention times for nucleotides in (A) and (B) are due to different buffers used for the preparation of isolated Snu114^72-1008^ and for Snu114^72-1008^–Prp8^SBR^ complexes.

We purified wt Snu114^72-1008^ alone and Snu114^72-1008^ variants in complex with Prp8^SBR^ variants and identified the bound nucleotide by reverse-phase ion-pair high-performance liquid chromatography (RP-HPLC). In all preparations, GTP was the only nucleotide detectable (Figure 3A and B). Furthermore, all mutant complexes contained GTP at comparable levels as wt Snu114^72-1008^ or the wt Snu114^72-1008^–Prp8^SBR^ complex (Figure 3A and B). These observations corroborate the idea that Snu114^72-1008^ has a strong intrinsic tendency to adopt a conformation that stably traps bound GTP. Based on our structure, stable GTP binding by Snu114^72-1008^ seems to be further supported by Prp8^SBR^.

**Figure 4.**
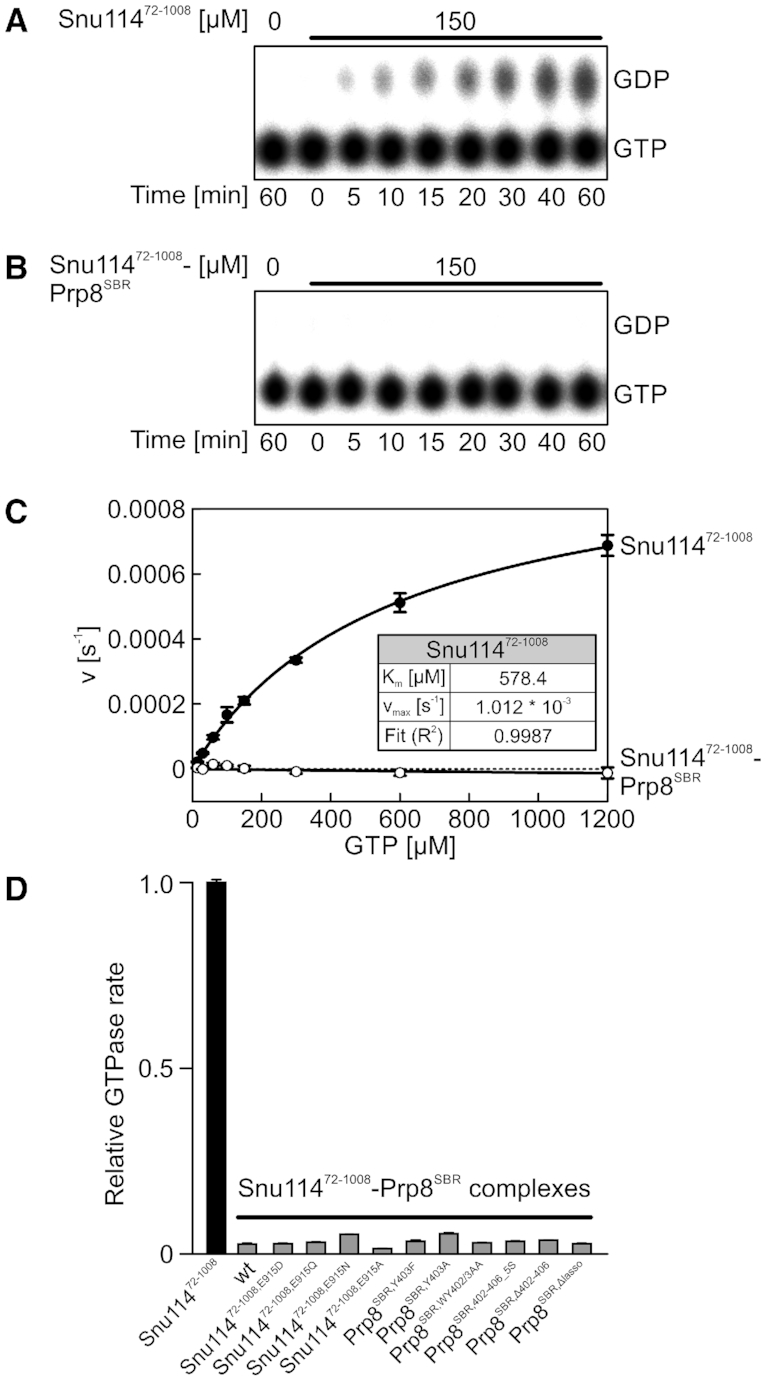
GTPase activities. (**A** and**B**) TLC monitoring time courses of GTP hydrolysis by Snu114^72-1008^ (A) or by the Snu114^72-1008^–Prp8^SBR^ complex (B). (**C**) Michaelis–Menten titrations of GTP hydrolysis by Snu114^72-1008^ or in the Snu114^72-1008^–Prp8^SBR^ complex. Inset—*K*_m_ and *v*_max_ of GTP hydrolysis by Snu114^72-1008^. Prp8^SBR^ leads to complete inhibition of the low intrinsic GTPase activity of Snu114^72-1008^. (**D**) Relative GTPase rates of Snu114^72-1008^ and of the indicated Snu114^72-1008^–Prp8^SBR^ complexes. Values in (C and D) represent means ± SD for three independent experiments.

To test the effect of Prp8 on Snu114 GTPase activity, we monitored Snu114^72-1008^ GTPase activity in isolation or in complex with Prp8^SBR^. To this end, we incubated Snu114^72-1008^ alone or in complex with Prp8^SBP^ for extended times at 30°C in the presence of α-[^32^P]-GTP, and monitored product nucleotides by TLC. Consistent with non-hydrolyzed GTP co-purifying with Snu114^72-1008^ or Snu114^72-1008^–Prp8^SBR^ complexes, very weak GTP hydrolysis was detectable under these conditions with Snu114^72-1008^ alone (Figure 4A). The weak intrinsic GTPase activity was completely abrogated by Prp8^SBR^ (Figure 4B). Michaelis–Menten titrations revealed a *K*_m_ of 578.4 μM and a *k*_cat_ of 1.012 * 10^−3^ s^−1^ for GTP hydrolysis by isolated Snu114^72-1008^ (Figure 4C).

Conversion of Prp8 Y403 to a phenylalanine or alanine, exchange of Snu114 E915 (contacting Prp8 Y403) to a glutamine, asparagine or alanine, deleting the Prp8 segment spanning the Snu114 nucleotide binding pocket (Prp8^Δ402–406^) or replacing it with five serines (Prp8^402–406_5S^), weakening the anchoring of this segment on Snu114 by exchange of Prp8 W402 (Prp8^W402A^) or of W402 and Y403 (Prp8^WY402/3AA^), or short-circuiting the neighboring Prp8 lasso structure (Prp8^Δ421–468^) would all be expected to interfere with the apparent Prp8^SBR^-mediated inhibitory mechanism targeting Snu114^72-1008^ H218, either by directly modulating Prp8 contacts to H218 or by weakening neighboring Snu114^72-1008^–Prp8^SBR^ interactions. However, none of the Snu114^72-1008^ or Prp8^SBR^ variants in the context of the Snu114^72-1008^–Prp8^SBR^ complex led to increased GTP hydrolysis (Figure 4D). These results suggest that Prp8^SBR^ inhibits Snu114^72-1008^ GTPase activity on several levels, including by sequestering H218 but also presumably by restricting hydrolysis-relevant conformational changes in Snu114^72-1008^.

### Effects of Snu114 and Prp8 variants on splicing

**Figure 5.**
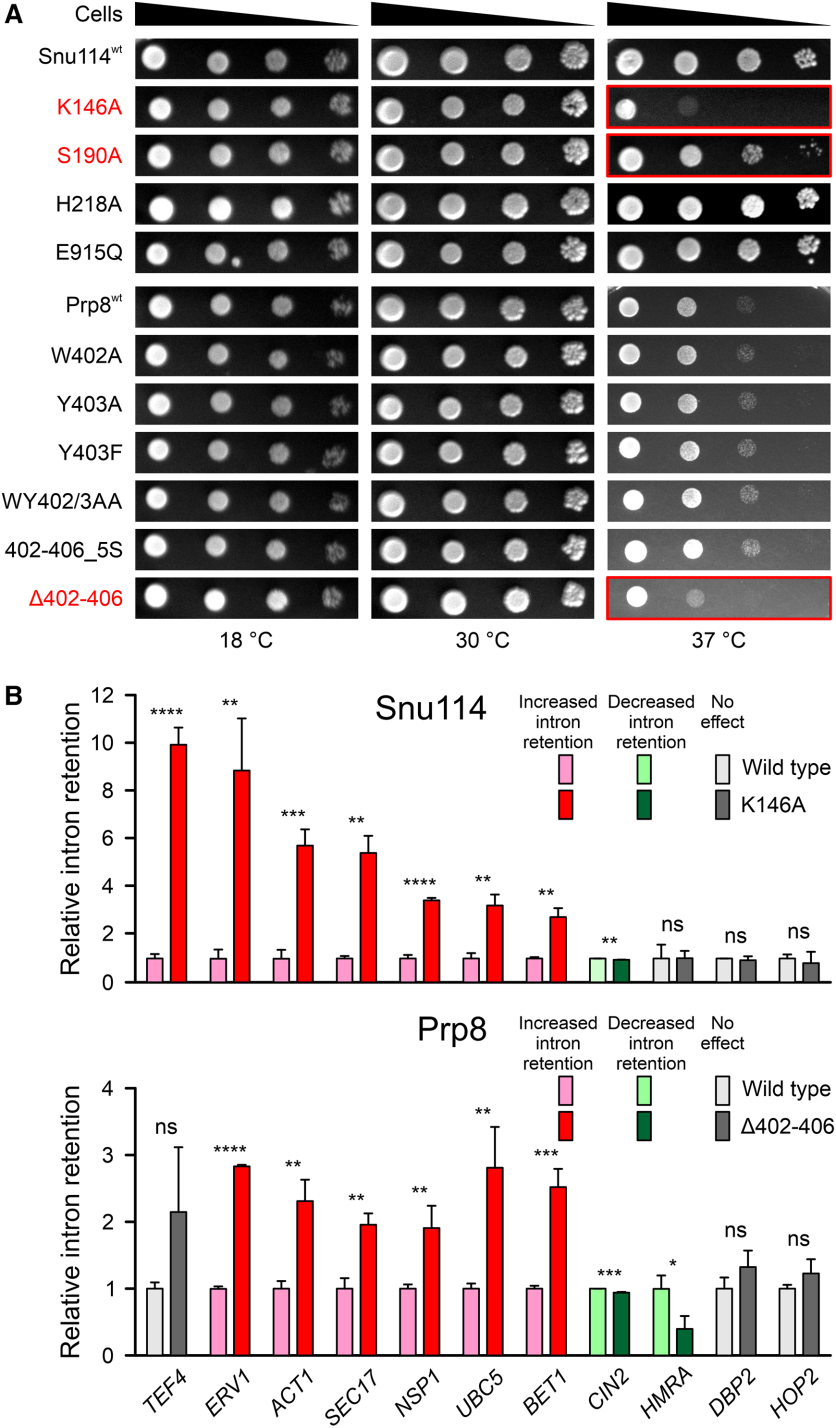
Yeast growth and *in vivo* splicing assays. (**A**) Serial dilutions (1, 10^−1^, 10^−2^, 10^−3^) of the indicated yeast strains, incubated at the indicated temperatures. Strains producing Snu114^K146A^ or Prp8^Δ402–406^ as the only Snu114 or Prp8 variants show mild temperature-sensitive growth (red). Colonies for each combination of target protein variants and temperature were grown on the same plate, and whole-plate images were uniformly adjusted for brightness and contrast. Serial dilutions were afterward separated into individual panels for display purposes. (**B**) Intron retention observed for the pre-mRNAs indicated at the bottom in strains producing Snu114^K146A^ (top) or Prp8^Δ402–406^ (bottom) relative to strains expression the respective wt protein. Snu114^K146A^ and Prp8^Δ402–406^ (dark colors) lead to increased intron retention (red) or decreased intron retention (green) in almost the same sets of genes relative to strains producing wt Snu114 or Prp8 (light colors). Values represent means ± SD for biological triplicates and technical duplicates. Significance indicators: ****, *P* ≤ 0.0001; ***, *P* ≤ 0.001; **, *P* ≤ 0.01; *, *P* ≤ 0.05; ns, not significant (*P*-values were calculated using Student's unpaired *t*-test).

We further investigated the possible functional role of the Snu114–GTP–Prp8 interaction network characterized above. To this end, we introduced plasmids that guided the expression of full-length Snu114 or Prp8 variants into yeast strains, carrying a sole copy of the *SNU114* or *PRP8* wt genes on counter-selectable plasmids, and monitored effects on yeast growth and splicing after eliminating the wt proteins. Snu114^T147A^ (exchange of a residue contacting the GTP β-phosphate) and Prp8^Δ421–468^ (bearing a deletion of the lasso-like region interacting with Snu114) did not support cell viability. Snu114^K146A^ (exchange of a residue contacting the GTP β and γ-phosphates), Snu114^S190A^ (exchange of a residue that coordinates Mg^2+^ and contacts the GTP γ-phosphate) and Prp8^Δ402–406^ (Snu114 H218-binding region) variants led to growth defects at 37°C (Figure 5A). Strains expressing the Snu114^H218A^ variant, in which the potential catalytic H218 is exchanged, or Snu114^E915Q^, in which a residue in the H218 interaction network is altered, showed no obvious growth differences compared to the parent strain. Consistently, lack of a growth phenotype upon H218 exchanges was noted before (17). Similarly, Prp8^Y403A^, Prp8^Y403F^, Prp8^W402A^, Prp8^WY402/3AA^ or Prp8^402–406_5S^ variants, in which residues contacting Snu114 H218 or the region running along the GTP-binding pocket of Snu114 are altered, did not lead to altered growth under the conditions tested.

To further delineate the basis of the growth defects observed at 37°C with Snu114^K146A^ and Prp8^Δ402–406^, we tested the splicing of a set of 11 pre-mRNAs (*TEF4*, *ERV1*, *ACT1*, *SEC17*, *NSP1*, *UBC5*, *BET1*, *HMRA*, *CIN2*, *DBP2* and *HOP2*) by quantitative real time (qRT) PCR in *snu114*_K146A and *prp8*_Δ402–406 strains, grown at the non-permissive temperature, compared to the parent strains. These pre-mRNAs have previously been used to assess splicing defects originating from yeast *prp8* mutations that are linked to retinitis pigmentosa in humans (37) or from mutations that lead to N-terminal truncations in the Brr2 helicase (38). Besides canonical introns, the collection includes pre-mRNAs with unusual 5′SS (*HOP2*), BP (*ERV1*, *CIN2*) or 3′SS (*SEC17*, *UBC5*), a pre-mRNA with an unusually short distance between the BS and 3′SS (*HMRA*) and a pre-mRNA with an unusually long intron (*DBP2*). Relative to the parent strains, intron retention of seven pre-mRNAs (*TEF4*, *ERV1*, *ACT1*, *SEC17*, *NSP1*, *UBC5*, *BET1*) was strongly increased in the *snu114*_K146A strain, intron retention was to a small but significant extent decreased in *CIN2*, while splicing of the other pre-mRNAs was unaffected (Figure 5B, top). Strikingly, in the *prp8*_Δ402–406 strain, intron retention for the same subset of pre-mRNAs was also increased, albeit to a smaller extent and intron retention was decreased again for *CIN2* and, in addition, for *HMRA* pre-mRNA (Figure 5B, bottom). For the same strains grown at a permissive temperature (30°C), or for strains expressing Snu114^H218A^ or Prp8^Y403A^, which did not show any growth defects, no effect on splicing of selected pre-mRNAs (*ERV*, *NSP1*) was observed (Supplementary Figure S2). While we did not observe a particular feature in the introns that correlates with increased retention upon altering Snu114 or Prp8, the four targets that do not show increased intron retention (*CIN2*, *HMRA*, *DBP2*, *HOP2*) all exhibit unusual, albeit diverse (unusual BS, short BS-3′SS distance, long intron, unusual 5′SS, respectively) features. On the one hand, these analyses indicate that altered pre-mRNA splicing elicited by certain Snu114 or Prp8 variants underlies growth phenotypes observed in corresponding mutant strains. On the other hand, they clearly suggest that the Snu114–GTP–Prp8 interaction network characterized here is important for at least a large subset of splicing events, possibly affecting all splicing events that involve introns with canonical features.

## DISCUSSION

Here, we have delineated a crystal structure of a large portion of the yeast Snu114 protein, containing all EF-G/eEF2-homologous regions, in complex with GTP and an intrinsically disordered Snu114-binding region of the Prp8 protein, investigated the nucleotide binding and hydrolysis activities of wt Snu114 alone and in complex with Prp8^SBR^, and of variants of this complex bearing exchanges in Snu114 or Prp8 residues that are expected to weaken Snu114–GTP or Snu114–Prp8 interactions. Moreover, we have monitored growth and splicing in yeast strains that harbored corresponding Snu114 or Prp8 variants as the only variants of the proteins.

Our structural analysis showed that Snu114 only complexed to Prp8^SBR^ adopts the same GTP-bound conformation as has so far been observed in all structures of Snu114/Prp8-containing spliceosomal complexes. Thus, Snu114–GTP–Prp8^SBR^ seems to constitute a rigid building block of the spliceosome, except perhaps for the C-terminal 43 residues of Prp8^SBR^, which change conformation in the isolated complex compared to the situation in spliceosomes. The conformational rigidity of the Snu114–GTP–Prp8^SBR^ unit may be additionally supported by a disulfide bridge that connects two parts of the Snu114 G domain and thereby provides intra-molecular cross-strutting. Although the reducing environment of the nucleus disfavors disulfide bridge formation, disulfide bridges have been observed in structures of nuclear proteins (39) and have been implicated in the function of some nuclear factors (40). The lack of major structural changes in the Snu114^72-1008^–Prp8^SBR^ sub-complex when studied outside the spliceosome is in stark contrast to conformational changes observed in the closely related translation factors EF-G/eEF2 (41,42), suggesting that Snu114 in the spliceosome exhibits a different mode of action or function compared to EF-G/eEF2 on the ribosome.

We find very low intrinsic GTPase activity associated with Snu114, as is the case for many G proteins including EF-G/eEF2 (43). Still, this activity could be reliably quantified and we unequivocally showed that it is completely abrogated in the presence of Prp8^SBR^. The latter observation formally establishes Prp8 as a GTPase-inhibiting protein, and thus to the best of our knowledge as the first GTPase-regulatory factor, of Snu114. As the conformation and nucleotide-bound state of the Snu114–GTP–Prp8^SBR^ sub-complex remains constant in all Snu114-containing yeast or human spliceosomal complexes structurally analyzed to date, Prp8 may constitute the sole Snu114 GTPase-regulatory protein in the spliceosome. Proteins or RNAs that serve as Snu114 GTPase-activating protein, G-nucleotide exchange factor of G-nucleotide exchange inhibitor, as have been found to regulate the activity cycles of small G proteins, may not exist in the spliceosome. It remains to be seen whether other proteins or RNAs might intermittently activate Snu114 GTPase, e.g. during U5 snRNP or U4/U6•U5 tri-snRNP assembly.

While presently, we have no evidence that Prp8-mediated shutdown of Snu114 GTPase activity *per se* has major consequences for splicing, we interpret our findings as another indication for Prp8^SBR^ stabilizing the Snu114 conformation, exerted by Prp8^SBR^ inter-connecting several domains of Snu114 and by locking GTP in a hydrolysis-resistant fashion inside of the Snu114 G domain. This notion is further supported by our observation that Snu114 variants, in which GTP-contacting residues are exchanged, are not expressed as soluble proteins in insect cells, but that their soluble expression can be rescued by co-production of Prp8^SBR^. We did not discern obvious structural features of Snu114 that could explain why it requires a bound GTP for stability when related G proteins do not. Answering this question would require, e.g. elaborate, comparative molecular dynamics simulations.

Also the magnitudes of the growth defects we observed upon mutating Snu114 or Prp8 in a manner that is expected to affect GTP binding or Prp8-Snu114 interactions are in line with the notion of a stable Snu114–GTP–Prp8^SBR^ sub-complex as a functional unit in the spliceosome. Only the deletion of the entire Prp8^SBR^ lasso region led to loss of cell viability. Other tested Snu114 or Prp8 variants either did not elicit a growth defect or led to mild growth defects at an elevated temperature. In these variants individual contact points or a small portion of the large Snu114–GTP or Snu114–Prp8^SBR^ contact regions were altered, and our co-purification studies clearly indicate that these variants do not lead to complete disintegration of the Snu114^78-1008^–GTP–Prp8^SBR^ complex. Rather, we suggest that the mutations destabilize local interactions with GTP or Prp8, rendering Snu114 more malleable, in particular at increased temperature.

Contrary to previous hypotheses that Snu114 might act as a molecular motor or as a regulatory G proteins during splicing, our results are consistent with the idea that Snu114 binds but does not hydrolyze GTP during a splicing cycle, in line with a similar previous suggestion (17). Rather, our findings suggest that the Snu114–GTP–Prp8^SBR^ sub-complex represents a stable building block of the spliceosome. This building block may serve as a binding platform that supports factor exchange or repositioning during a splicing cycle. Indeed, focusing on the Snu114–Prp8^SBR^ region, a number of other proteins transiently bind to this region during a splicing process (Supplementary Figure S3). Strikingly, the sets of proteins bound at the Snu114–Prp8^SBR^ region change from the B to the B^act^ complex and again in the P and ILS complexes (Supplementary Figures S3), i.e. precisely during the stages of a splicing cycle (activation and disassembly, respectively), in which Snu114 has been implicated (14). As the Snu114–GTP–Prp8^SBR^ sub-complex clearly provides a key landing pad for transiently integrated spliceosomal factors, and as the newly incoming factors help propel the spliceosome along the splicing pathway, we consider Snu114–Prp8 as a ‘relay station’ that enables efficient splicing.

Taken together, our results suggest that in the context of the spliceosome, Snu114 has been converted into a pseudo-GTPase, at least in part due to stable interaction with Prp8^SBR^, which now serves as a rigid landing pad for other splicing factors, and which thereby might facilitate specific transitions in a splicing cycle. The suggested mechanism of Prp8 converting Snu114 into a stable scaffold, on which other factors can assemble, which involves fixing GTP in its binding site while preventing hydrolysis, is reminiscent of the core of the exon junction complex. In the latter case, the MAGOH and Y14 proteins lock the ATP-bound DEAD-box RNA helicase eIF4AIII in a pre-hydrolytic state on RNA (44,45). eIF4AIII is thereby transiently reprogrammed from an RNA/RNP remodeling enzyme to a scaffold protein that allows the build-up of a larger RNA-protein complex.

## DATA AVAILABILITY

Structure factors and coordinates have been deposited in the RCSB Protein Data Bank (https://www.rcsb.org/) with accession code 6TEO and will be released upon publication.

## Supplementary Material

gkaa182_Supplemental_File

## Notes

Present address: Oleg M. Ganichkin, Proteros Biostructures GmbH, Bunsenstraße 7a, D-82152 Martinsried, Germany.

Present address: Eva Absmeier, MRC Laboratory of Molecular Biology, Cambridge Biomedical Campus, Francis Crick Avenue, Cambridge CB2 0QH, UK.
