# Supplementary material for: A Snu114–GTP–Prp8 module forms a relay station for efficient splicing in yeast
Source: Nucleic Acids Res. 2020 Mar 20;48(8):4572–84. doi: 10.1093/nar/gkaa182 (PMC7192624; doi:10.1093/nar/gkaa182)
Supplement: gkaa182_Supplemental_File [file gkaa182_supplemental_file.pdf]

SUPPLEMENTARY DATA

A Snu114-GTP-Prp8 module forms a relay station for efficient splicing in yeast

Junqiao Jia[1,†], Oleg M. Ganichkin[1,3,†], Marco Preußner[2], Eva Absmeier[1,4], Claudia Alings[1], Bernhard Loll[1], Florian Heyd[2], Markus C. Wahl[1,5,*]

[1] Freie Universität Berlin, Laboratory of Structural Biochemistry, Takustraβe 6, D-14195 Berlin, Germany

[2] Freie Universität Berlin, Laboratory of RNA Biochemistry, Takustraβe 6, D-14195 Berlin, Germany

[3] Present address: Proteros Biostructures GmbH, Bunsenstraße 7a, D-82152 Martinsried, Germany

[4] Present address: MRC Laboratory of Molecular Biology, Cambridge Biomedical Campus, Francis Crick Avenue, Cambridge, CB2 0QH, United Kingdom

[5] Helmholtz-Zentrum Berlin für Materialien und Energie, Macromolecular Crystallography, Albert-Einstein-Straße 15, D-12489 Berlin, Germany

[†] These authors contributed equally to this work.

* Correspondence to: markus.wahl@fu-berlin.de

SUPPLEMENTARY TABLES

Table S1. Primers[a] used for qRT-PCR.

Genes	Primer Sequence	Genes	Primer Sequence
*TEF4*_Ex1_F[a]	TGGGTCCAAAGGGCTTAAAG	*BET1*_Ex_F	TGTATCATCAGCGCTAGGGA
*TEF4*_Intr1_R	TGCCAAATAAACGAACGGGA	*BET1*_Ex_R	TCTCTGACCCATAGCTCCCA
*TEF4*_Ex2_R	ACGTCGGAACCTAATAAGCG	*BET1*_Intr_F	TTGACCACGAGTGAACTTCG
*ERV1*_Ex_F	GAACTTGGGAGGTGGATGTG	*BET1*_Intr_R	ACGTTGATAAGCGTTTCCCC
*ERV1*_Ex_R	GTCAAATTTGGGCTTCCTCA	*HMRA1*_Ex_F	AGAAAGCAAAGCCTTAATTCCA A
*ERV1*_Intr_F	CGCAAAGCAATTCAGGATCT	*HMRA1*_Ex_R	TGCCACATTTCTTTGCAACTTC
*ERV1*_Intr_R	CCCGGTCACGTACTGAAAGT	*HMRA1*_Intr_F	AGTTGCAAAGAAATGTGGCA
*ACT1*_Intr_F	CGCTTGCACCATCCCATTTA	*HMRA1*_Intr_R	ACATACCCAAA CTCTTACTTGAA
*ACT1*_Intr_R	AGGAGGTTATGGGAGAGTGAA	*CIN2*_Ex_F	TGTTGAACGTACGGAGCTTG
*ACT1*_Ex_F	CGAATTGAGAGTTGCCCCAG	*CIN2*_Ex_R	GCTGAATCACCCTTCTC CAA
*ACT1*_Ex_R	CGGCTTGGATGGAAACGTAG	*CIN2*_Intr_F	CTGCTTTCATTATGCGTCCA
*SEC17*_Ex_F	GCTGCTGATCTTTGTGTCCA	*CIN2*_Intr_R	TGGTCTTCTTCTGCAAGGTCT
*SEC17*_Ex_R	TCAGCAGCTTTCAAAAACGA	*DBP2*_Ex_F	AGGAGATCTTATGGTGGCGG
*SEC17*_Intr_F	GGGAATTGATATTTCCCGTTG	*DBP2*_Ex_R	AACGACCTCTGTTACCC CAG
*SEC17*_Intr_R	GATCAGCAGCCTCCTCAAA C	*DBP2*_Intr_F	AGTCGTTTTGAGAGACGGGA
*NSP1*_Ex_F	GCCTTCGGAACAGGTCAATC	*DBP2*_Intr_R	TCCTTGTTTATTTGCGCCGA
*NSP1*_Ex_R	TGTTCGTGTTATTTGGCGCA	*HOP2*_Ex_F	AAACAACAGCAACCAAAGCA
*NSP1*_Intr_F	AACGAAAACCTGCGAACCTG	*HOP2*_Ex_R	CGAAGGTTTTAGACACTATGCGT
*NSP1*_Intr_R	GGGCGTTTTGTTTTGTTGAGG	*HOP2*_Intr_F	ACATGCTCATCAAATACCGC C
*UBC5*_Ex_F	TTTGGTCCCTGAAATTGCTC	*HOP2*_Intr_R	CAGCTGATAAAATACCCGGGG
*UBC5*_Ex_R	GCGGTGGCTTCATACTTAGC		
*UBC5*_Intr_F	AAATGAATGATTTTTCTTGATCTG		
*UBC5*_Intr_R	AGGCTTGCCAATGATACAGG		
*HOP2*_Intr_R	CAGCTGATAAAATACCCGGGG		

[a] Primer sequences from (37) and (38).
[b] Ex, exon; Intr, intron; F, forward; R, reverse.

SUPPLEMENTARY FIGURES

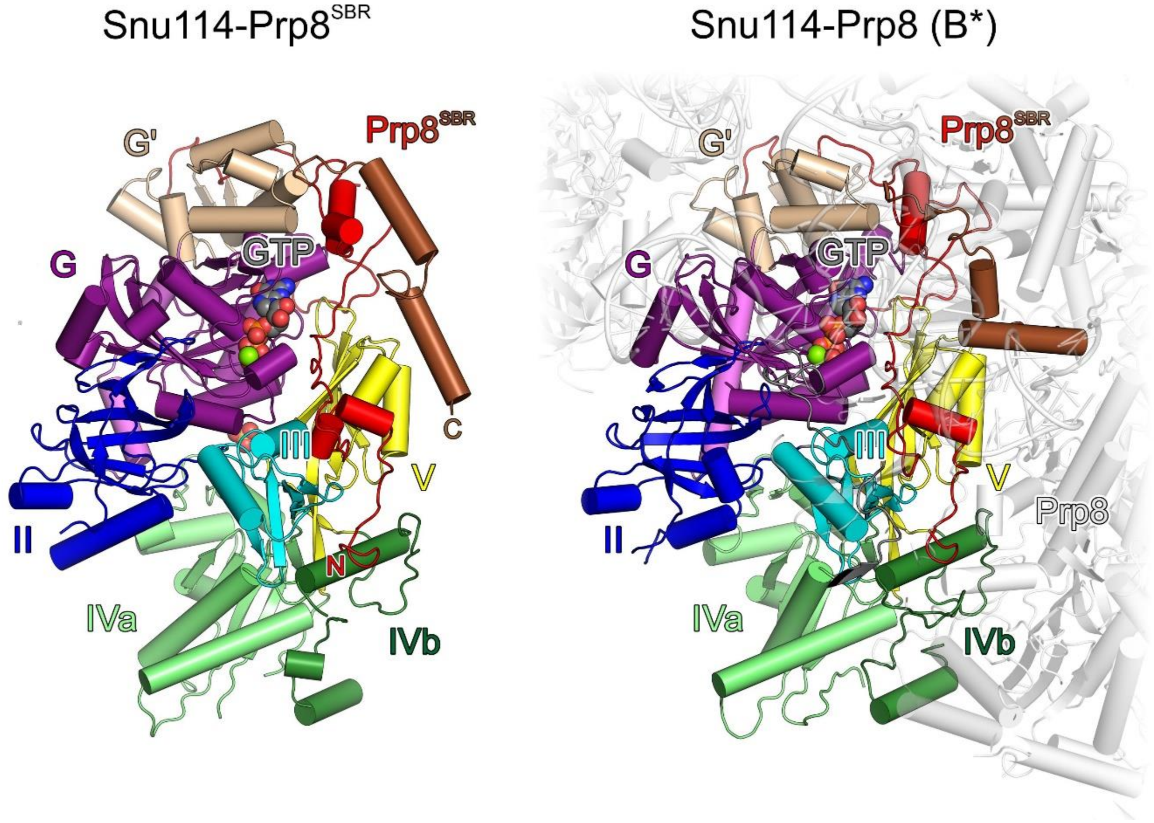

Figure S1. Comparison of the Snu114$^{72\text{-}1008}$-Prp8^{SBR} complex in isolation (left) and in the context of the yeast spliceosomal B* complex (right; PDB ID 6J6G) (50). The 43 C-terminal residues of Prp8^{SBR} (brown) are folded differently in isolated complex and in the spliceosome.

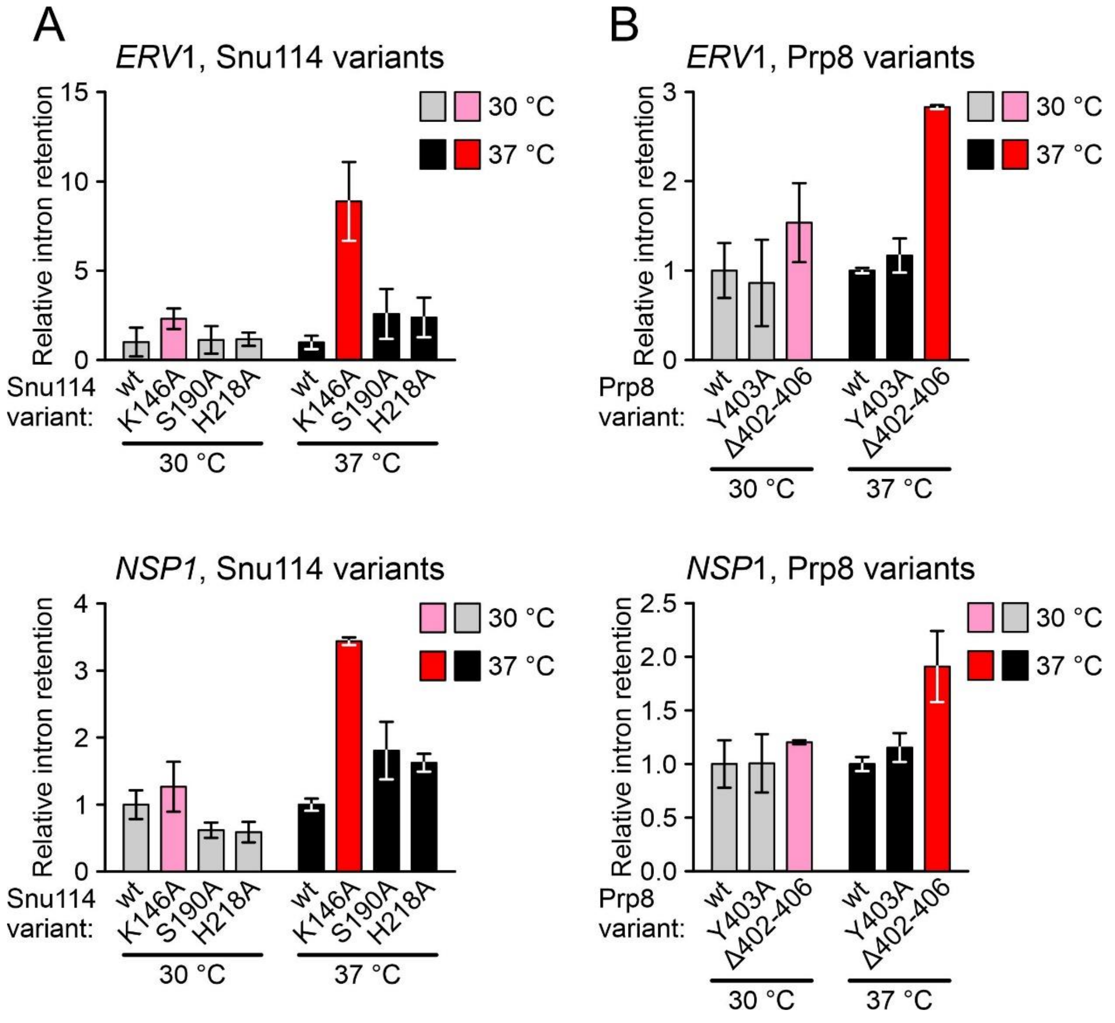

Figure S2. *In vivo* splicing assays. **(A,B)** Intron retention observed for *ERV1* (top) and *NSP1* (bottom) pre-mRNAs in strains producing the indicated Snu114 **(A)** or Prp8 **(B)** variants, relative to the respective pre-mRNAs in strains with wt Snu114/Prp8, at 30 °C (light bars) and at 37 °C (dark bars). Bars for variants that exhibit growth defects at 37 °C are marked in light red (30 °C) and red (37 °C). Values represent means ± SD for biological triplicates and technical duplicates.

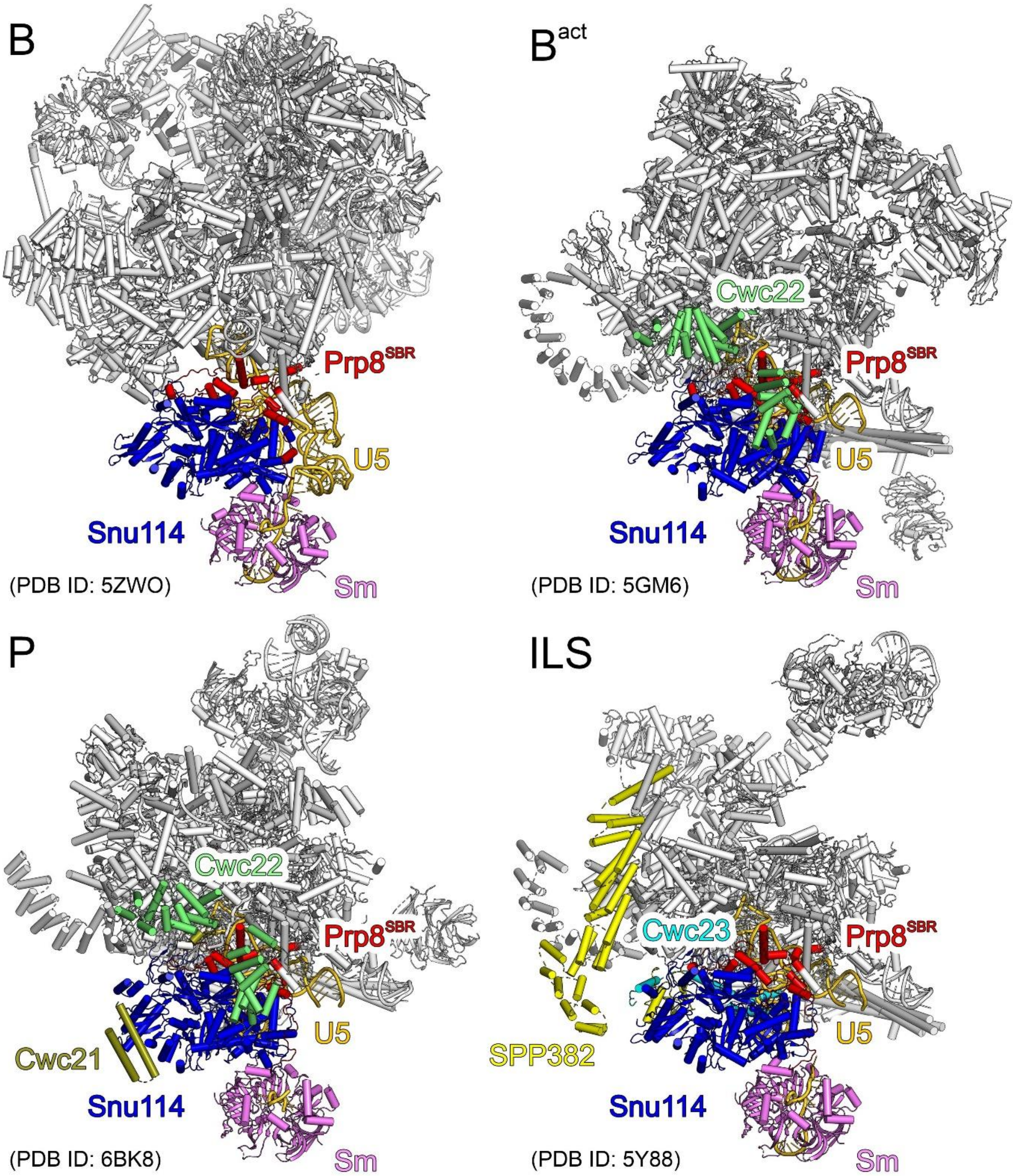

Figure S3. A Snu114-GTP-Prp8 module as a relay station during splicing. Structures of yeast B complex (PDB ID: 5ZWO) (48), B^{act} complex (PDB ID: 5GM6) (49), P complex (PDB ID: 6BK8) (53) and ILS (PDB ID: 5Y88) (54) (complexes identified in the upper left corners) after superposition *via* their Snu114 subunits. Snu114 (blue), $Prp8^{SBR}$ (red), U5 snRNA (gold) and the U5 Sm core domain (violet) are highlighted in each structure. The structure of the Snu114-GTP-$Prp8^{SBR}$ module hardly changes in the different complexes depicted. Snu114-GTP-$Prp8^{SBR}$ serves as binding platform for different proteins (additionally highlighted and labeled)

in the spliceosomal complexes depicted. The remaining RNA and protein subunits of the complexes are shown in gray.
